# A Dyadic Action Control Trial in Overweight and Obese Couples (DYACTIC)

**DOI:** 10.1186/1471-2458-14-1321

**Published:** 2014-12-24

**Authors:** Urte Scholz, Corina Berli

**Affiliations:** Department of Psychology, University of Zurich, Applied Social Psychology, Binzmuehlestrasse 14 / Box 14, CH-8050 Zürich, Switzerland; Institute of Psychology, University of Bern, Health Psychology, Fabrikstrasse 8, CH-3012 Bern, Switzerland

**Keywords:** Physical activity, Randomized controlled trial, Action control, Couples, Health Action Process Approach, Dyadic

## Abstract

**Background:**

Enhancing physical activity in overweight and obese individuals is an important means to promote health in this target population. The Health Action Process Approach (HAPA), which was the theoretical framework of this study, focuses on individual self-regulation variables for successful health behavior change. One key self-regulation variable of this model is action control with its three subfacets awareness of intentions, self-monitoring and regulatory effort. The social context of individuals, however, is usually neglected in common health behavior change theories. In order to integrate social influences into the HAPA, this randomized controlled trial investigated the effectiveness of a dyadic conceptualization of action control for promoting physical activity.

**Methods/Design:**

This protocol describes the design of a single-blind randomized controlled trial, which comprises four experimental groups: a dyadic action control group, an individual action control group and two control groups. Participants of this study are overweight or obese, heterosexual adult couples who intend to increase their physical activity. Blocking as means of a gender-balanced randomization is used to allocate couples to conditions and partners to either being the target person of the intervention or to the partner condition. The ecological momentary intervention takes place in the first 14 days after baseline assessment and is followed by another 14 days diary phase without intervention. Follow-ups are one month and six months later. Subsequent to the six-months follow-up another 14 days diary phase takes place.

The main outcome measures are self-reported and accelerometer-assessed physical activity. Secondary outcome measures are Body Mass Index (BMI), aerobic fitness and habitual physical activity.

**Discussion:**

This is the first study examining a dyadic action control intervention in comparison to an individual action control condition and two control groups applying a single-blind randomized control trial. Challenges with running couples studies as well as advantages and disadvantages of certain design-related decisions are discussed. This RCT was funded by the Swiss National Science Foundation (PP00P1_133632/1) and was registered on 27/04/2012 at http://www.isrctn.com/ISRCTN15705531.

**Electronic supplementary material:**

The online version of this article (doi:10.1186/1471-2458-14-1321) contains supplementary material, which is available to authorized users.

## Background

Regular physical activity has beneficial effects on health [1, 2] just as physical inactivity was identified the fourth leading risk factor for mortality [2]. Current recommendations of the World Health Organization on physical activity for adults are to engage in at least 150 minutes of moderate-intensity aerobic activity or 75 minutes of vigorous-intensity aerobic activity or a combination thereof. The activity can be broken down into several bouts of at least ten minutes duration. Moreover, muscle-strengthening activities on two or more days a week are recommended. There is, however, a discrepancy between these recommendations and the actual physical activity in the adult population worldwide in general [3] as well as in Switzerland in particular [4]. Of the general Swiss adult population 28% do not meet these minimum requirements [4]. Especially for overweight and obese individuals, regular physical activity is highly recommended for weight regulation and health benefits [5]. Prevalence data for overweight or obesity (BMI > = 25) in Switzerland report on 41% in the population older than 15 years [4]. Thus, enhancing physical activity in overweight and obese individuals is of special importance.

Changing one’s unhealthy behavior, however, seems to be a major challenge for most people. This is even the case when individuals report strong intentions to change their behavior: By far not all individuals having a strong intention to change a behavior are successful in doing so, a phenomenon that is known as the intention-behavior gap [6]. There is a plethora of research on health behavior change which is usually based on one of the leading social-cognitive models of health behavior, such as the Theory of Planned Behavior (TPB) [7], the Protection Motivation Theory (PMT) [8], Social Cognitive Theory (SCT) [9], or the Health Action Process Approach (HAPA) [10]. Whereas the TPB, SCT, and PMT assume intentions to be the most important predictor of behavior, the HAPA is one of the few models that explicitly take the intention-behavior gap into account. Studies on physical activity applying the HAPA have demonstrated the model’s utility for this specific health behavior [11–13].

The HAPA distinguishes between a motivational and a volitional phase. In the motivational phase, risk awareness (i.e., the perceived personal health risk due to the unhealthy behavior that needs to be changed), positive and negative outcome expectancies (i.e., perceived advantages and disadvantages of a behavioral change; [14]) and self-efficacy (i.e., the perceived competence to change the behavior despite obstacles; [14]) are assumed to predict intentions. In the volitional phase, in addition to intentions and self-efficacy, volitional predictors are specified to explain behavior. These are action planning (i.e., when, where, and how to implement the intended behavior; [15–17]), coping planning (i.e., anticipating barriers to the intended behavioral change and planning how to overcome these barriers; [18]) and action control. Action control is based on the concept of feedback loops from the self-regulation theory by Carver and Scheier [19] and comprises three subcomponents: awareness of standards, self-monitoring, and self-regulatory effort [20]. Awareness of standards is comparable to the standard value in feedback loops, but refers not only to setting an intention but also to remembering this intention in situations important for the respective self-regulatory action. Without being aware of one’s own standards/behavioral intentions, successful self-regulation cannot be accomplished. Self-monitoring refers to the attentive monitoring of qualitative and quantitative aspects of one’s own behavior. Research on self-monitoring has identified this component to be of great importance for successful self-regulation e.g., [21, 22]. In the concept of action control, self-monitoring is also assumed to trigger the comparison between the intended and the actual behavior [23]. If a discrepancy between a person’s standard (e.g., behavioral intention to eat five portions of fruits and vegetables a day) and his/her actual behavior (e.g., having eaten only two portions of fruits and vegetables today) is detected, action must be taken. This is the function of the third component, self-regulatory effort: Appropriate means of reducing the discrepancy between actual and intended behavior must be applied [20]. Action control has been demonstrated to be effective in translating intentions into behavior in both correlational [20, 24] and experimental studies [23, 25].

There are several experimental means to foster action control. Two behavior change techniques are directly related to action control: “self-monitoring of behavior” and “discrepancy between current behavior and goal” [26]. Moreover, one approach that has, however, not yet been linked explicitly to action control is the use of reminders, as these address not only the awareness of intentions, but are also assumed to stimulate self-monitoring and (indirectly) self-regulatory effort. Research on the effects of reminders is very common in the context of adherence e.g., [27, 28]. Studies on reminder systems – either with technology support like online-pagers or simple telephone or mail reminders - provide good evidence that this is a useful tool to promote the implementation of an intended behavior. Likewise, there are some studies on mobile phone based text message reminders demonstrating the effectiveness of these interventions [29].

### Dyadic approaches to health behavior change models

Individuals usually try to change their health behavior (or refrain from doing so) while being embedded in a social network of partnership, family, friends and colleagues. Almost no health behavior change theory, however, takes the social context explicitly into account^a^. Instead the focus usually is on individual self-regulation. One approach to include social exchange processes into health behavior change models is to add the most prominent ones, social support and social control, as predictors [31–33]. A recent alternative attempt to introduce a social component into health-behavior change research is the dyadic conceptualization of originally individual components of health behavior change models that have been shown to be of importance for successful behavioral change on the individual level. One such approach is the concept of collaborative/dyadic planning with a partner [34–36]. For example, Prestwich and colleagues [35] investigated the effect of collaborative action planning on physical activity, when plans were formed *and* enacted together with a partner. The authors found that all participants who planned collaboratively were more successful in increasing their physical activity than individual planning or control group participants. Moreover, Burkert and colleagues [34] found that action control and social control mediated the effects of a dyadic planning intervention. What has not yet been targeted on the dyadic level, however, is action control. This was the main aim of the present study. Targeting dyadic action control can be done by focusing on the partner as the provider of text messages addressing the behavior-specific components of action control (self-monitoring, awareness of intentions, self-regulatory effort) for the target person. Applying an experimental manipulation of dyadic action control provides a strong test for dyadic influences in behavior change. Moreover, it can also considerably further our knowledge on the role of social exchange processes within a health-behavior change framework as enhanced levels of social control and social support are the assumed main mediating mechanisms of a dyadic action control intervention. Furthermore, interactions between individual self-regulation abilities and experimental conditions can be examined in order to further test the hypothesis that social regulation might compensate for deficits in individual regulation capacities on an experimental basis. To the best of our knowledge, this has not yet been done.

### Intra- and interpersonal level of analysis

Although all theories of health behavior change postulate time-dependent processes occurring within individuals, the vast majority of studies analyze the data on the between-subjects level. Associations at the within (intrapersonal) or between (interpersonal) level, however, can differ substantially [37]. As a consequence, a stringent test of our theories will always require testing the theory on both levels of analysis. A related criticism on standard RCTs is that the level of (longitudinal) assessment is usually on a macro-time-level (e.g., baseline and several weeks or months follow-up), which usually only allows interpersonal analyses, but not on a micro-time level (i.e., daily or weekly assessments) cf. [38] which would allow testing for intrapersonal associations. This means that, for example, a randomized controlled trial with baseline assessment, treatment and a follow-up assessment can answer questions on treatment effects (e.g., on physical activity) and on mediating processes at the between-person level (e.g., whether the treatment effect is achieved by increased self-efficacy induced by the treatment). It is not possible, however to answer questions on processes that take place in the time right after the intervention and within persons (e.g., the development of self-efficacy in relation to mastery experience on a daily or weekly basis; [39]).

This can be addressed by applying micro-time assessments. Micro-time assessment comprises weekly or daily diary data e.g., [40] or ambulatory momentary assessment (AMA) e.g., [41]. Studies are needed that combine assessments on macro-time and micro-time levels. For example, randomized controlled trial designs that aim at changing behavior on a macro-time level (i.e., six or twelve months after treatment) can be combined with micro-time assessments in the first phase after treatment. Another alternative is to apply an ecological momentary intervention (EMI), that is, “…treatments characterized by the delivery of interventions to people as they go about their daily lives” [42] in combination with momentary assessment and longer-time follow-ups. This allows capturing comprehensively the processes taking place during as well as (right) after dyadic and individual interventions during everyday life [42].

### Aims of the present study

The aims of the present study are fourfold. The first aim is to examine the effectiveness of a dyadic action control intervention in comparison to an individual action control condition and two control groups. Second, we aim at examining whether a dyadic action control intervention is especially beneficial for individuals low in their individual self-regulation capabilities. Third, mediating mechanisms of the dyadic and individual action control conditions will be examined and compared. The proposed design will allow examining micro- and macro-time changes in outcomes as well as potential mediating mechanism using a daily diary assessment together with objective assessment of the target behavior. Fourth, potential gender differences will be examined. For the hypotheses related to these aims, please see the trial registration: http://www.isrctn.com/ISRCTN15705531. This trial was registered on 27/04/2012.

## Methods/design

Participants of this trial are heterosexual couples, who live in a committed relationship for at least 12 months and cohabit for at least 6 months. Both partners have to be overweight or obese (BMI ≥ 25) and both need to be physically inactive and have the intention to change their physical activity patterns. These requirements are made because an action control intervention is assumed to be only beneficial in a postintentional sample [10]. Moreover, both partners should speak German fluently, be between 18 and 75 years old, and have the possibility to receive and read text messages throughout the day. Women should not be pregnant and both partners should not work in 24 h shift work and should not participate in a professional weight loss program.

**Figure 1:**
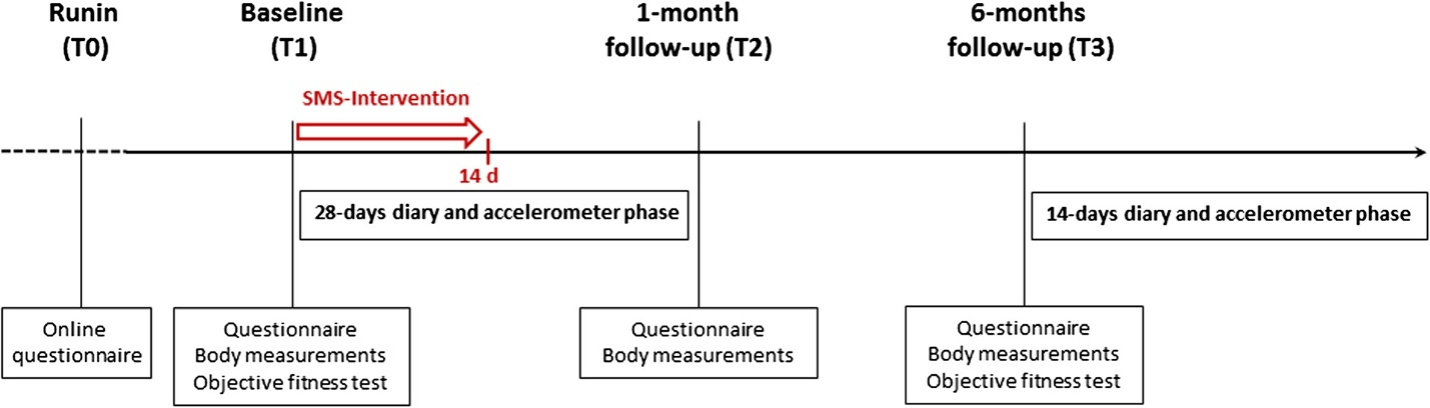
**Longitudinal design.**

This single-blind randomized controlled trial comprises a longitudinal design with micro- and macro-time assessments. Recruitment is organized via advertisements in newspaper and on webpages, flyer and postings in medical and sport facilities, public transport, local companies and mailings to private households in the city of Berne and surroundings, and a marketing research institution. Interested people can send an email to the study team or use a contact page on the homepage of the University of Berne. Participants are then contacted via telephone and inclusion and exclusion criteria are checked. In case of couples meeting all inclusion criteria, both partners are sent separate links for a short online-questionnaire (T0) for which participants provide informed consent. After both partners have completed the online questionnaire, participants are contacted again and are invited to the lab. If participants report having at least one health concern with regard to engaging in physical activity in the short online questionnaire, they are asked to contact a doctor prior to study participation and to check with the doctor whether or not participation for them is possible. At the baseline assessment (T1) participants receive full information on the study, provide informed consent and complete the baseline questionnaire and objective assessments. Moreover, the first part of the intervention for all but the control groups is implemented and they are introduced to the technical devices for the study (smartphones and accelerometers). The day after baseline, a 28 days diary and accelerometer phase starts (D1_1-D1_28). These 28 days comprise during the first 14 days the main part of the intervention for all but the control groups. One month after baseline the first follow-up (T2) takes place. Participants are again invited to come to the lab to return the smartphones and accelerometers and to complete another questionnaire and objective assessments. Six months later, the second follow-up takes place (T3), including again the completion of a questionnaire and objective assessments in the lab. Subsequently, a second 14 days smartphone-based diary and accelerometer phase takes place (D2_1 – D2_14) (see Figure 1 for the longitudinal design). All participating couples receive 200 Swiss Franks as a financial incentive for completing the study.

### Randomization

The randomization comprises on the one hand the randomization to one of the four groups (two control groups and two intervention groups) and on the other hand the gender-balanced randomization of the couple into a target person receiving the intervention and a partner condition. For this reason, blocking as means of restricted randomization is used. Within a block of eight participating couples, two couples each are assigned to one of the four groups, once the man and once the woman assigned as target person. Before the beginning of the study, a computerized random-number generator is used for sequence generation of blocks. This allocation sequence is generated by an assistant that is not part of the study team and concealed in a set of sealed, numbered envelopes, and thus remain unknown to any of the investigators until the group assignment. On the day of the baseline assessement (T1), the interviewer conducting the session opens the appropriate numbered envelope and prepares the study materials accordingly.

### Detailed description of intervention and control groups

This study applies an ecological momentary intervention [42] and comprises four groups: a dyadic action control intervention group, an individual action control intervention group, and two different control groups (see below).

**Figure 2:**
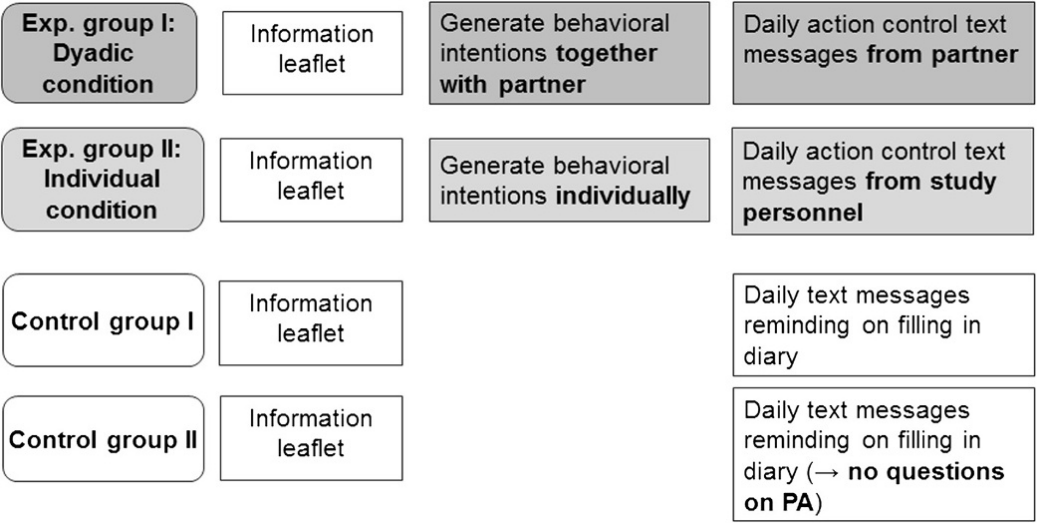
**Experimental design.** Note: Exp. group = experimental group; PA = physical activity.

At baseline assessment (T1), all participants (i.e. target persons and partners of all groups) receive an information leaflet on the benefits of moderate-to-vigorous physical activity for health and weight management and the recommendations on physical activity of the Swiss Federal Office of Sports (BASPO). The recommendation in 2011, when this study was planned, was to engage at least 30 minutes in moderate-to-vigorous physical activity per day. Subsequently, they are asked to answer a short quiz in order to make sure they understand the relevant information included in the leaflet. Wrong answers to this quiz are discussed with participants in order to provide correct information. This comprises the BCT “information about health consequences” [26]. In the following 14 days, all participants receive a short text message (SMS) once every weekday between 9–12 a.m. and 2–5 p.m. on their study smartphone (content of this SMS differs for intervention and control groups as well as for target persons and partners, see below). The time of the day the SMS is sent is randomly chosen, but equal for all study participants regardless of group membership. Figure 2 displays the experimental design.

#### Experimental group I: dyadic action control group

After completing the assessments at the baseline session, target persons and their partners are instructed to collaborately form behavioral intentions to increase the target person’s physical activity to the recommended levels (e.g., “get off the bus two stops earlier when going to work” or “take the bike instead of the car”). This is supervised by a trained interviewer in order to ensure the correctness of the behavioral intentions. In terms of BCTs this part of the intervention comprise goal setting (behavior) [26].

Partners are instructed to send daily standardized text messages aiming at increasing the target person’s physical activity-specific action control in personalized form (e.g., “Dear Peter, which of the planned activities have you already carried out today? Love, Regula”) on weekdays during the two weeks following baseline assessment. All action control messages were developed by the authors and reviewed with regard to their action control content by two external reviewers who are experts in the field. A full list of all ten messages can be found in the Additional file 1: Table S1. In terms of BCTs, the different messages target self-monitoring of behavior and discrepancy between current behavior and goal [26]. Moreover, some messages are simple reminders on the behavioral intentions, targeting the awareness of own standards. Partners are instructed to save the personalized text messages as drafts in their study smartphones at the baseline assessment under the supervision of the study interviewer, and receive a reminder text message from the study personnel every day instructing them to send the text message within an hour (and to fill in the diary at the end of the day). Target persons are not informed about the instructions given to the partner, and both partners are asked not to talk about the text messages during the ongoing diary phase.

Moreover, both target persons and partners are instructed not to delete any of these SMS from the smartphones as this will allow a control of the implementation of the study protocol by participants [43].

#### Experimental group II: individual action control group

After completing the assessments at the baseline session, target persons alone are instructed to form behavioral intentions to increase their physical activity to the recommended levels. This is supervised by a trained interviewer to ensure the correctness of the behavioral intentions. On weekdays during the two weeks following baseline assessment, the target persons receive a text message daily from the study personnel with the same physical activity-specific action control content as the experimental group I. Partners of the target persons in this condition receive a text message at the same time reminding them to complete the diary at the end of the day. Again, both target person and partners are instructed not to talk about the text messages and not to delete any of these SMS from the smartphones as this will allow a control of the implementation of the study protocol by participants [43].

#### Control group I: full diary version

Couples in control group I are not instructed to form any behavioral intentions, but also receive SMS (at the same time as all other participants) with the reminder to complete the end-of-day diary.

#### Control group II: diary without self-reported physical activity

Couples of this second control group receive the same instructions and reminder text messages as control group I participants. As completing a diary on self-reported physical activity might in itself trigger self-monitoring, albeit not as strongly as the two intervention groups, the second control group serves the purpose to control for this potential diary effect by only completing questions on social-cognitive variables, but not on self-reported physical activity. Thus, this group will rely on ambulatory momentary monitoring of physical activity by means of an accelerometer only.

### Measures

For all groups, the online questionnaire (T0) comprises measures on sociodemographics, habitual physical activity, current diseases and activity-related health risks, and participating couples’ relationships.

At baseline (T1) both partners complete a comprehensive questionnaire for baseline assessment on current physical activity, physical-activity specific HAPA-variables, social control and social support with regard to physical activity, indicators of relationship quality, indicators of well-being as well as control variables. Likewise, weight, height and waist, and hip circumference (to assess the waist-hip ratio) are objectively assessed from both partners. Moreover, an objective submaximal aerobic fitness test on a bicycle ergometer is conducted with the target person.

The subsequent daily diaries (D1_1 – D1_28) include short scales/single items on HAPA variables, social support, social control, relationship quality, indicators of subjective well-being and for all but the second control group participants, a self-report physical activity assessment. Moreover, during the 28-days diary phase, all participants (target persons and partners) wear triaxial accelerometers around the hip (GT3X+ monitor devices; ActiGraph, Pensacola FL).

At the first and second follow-up (T2 and T3), both partners again complete a comprehensive questionnaire comparable to the T1 questionnaire and weight, height and waist, and hip circumference (to assess the waist-hip ratio) are objectively assessed. Only at T3, the objective submaximal aerobic fitness-test on a bicycle ergometer is conducted again with the target person.

Right after the second follow-up, the second 14-days diary phase plus accelerometer-assessment of physical activity takes place in order to be able to have another objective assessment of physical activity plus the accompanying cognitions and feelings assessed in the diary. This diary phase is very similar to the first one, but without including an intervention component in the intervention groups.

### Statistical analyses

To test the central hypotheses for the intervention effects at the between-person level with the baseline and follow-up points of measurement, repeated measures ANOVAs will be conducted using SPSS. Mediator analyses for the intervention effects will be done by means of regression analyses (Preacher & Hayes, 2004, 2008). To analyze intervention effects at the between- and within-person level on a daily basis using the first diary phase, multilevel modeling will be used [44]. Frequency and duration of physical activity can be considered a count variable, thus, generalized linear mixed Poisson models with a logarithmic link function will used to analyze the data [45]. To test dyadic associations (e.g., during the diary phase), two-level longitudinal models will be fit.

### Power analysis

Effect sizes of action control interventions are medium to large (e.g., *f*^2^ = .25 in the study of Scholz & Sniehotta [46]; *f*^2^ = .42 in the study of Schüz et al. [23], for the volitional group). Thus, the analyses for the intervention effects from baseline to the two follow-ups with an assumed small correlation among repeated measures of *r* = .2 due to the expected change across time, with a power of .80, an alpha level of .05, a medium effect size of *f*^2^ = .25, and four intervention groups requires a total sample size of *N* = 88 couples (analyses are powered for between-effects on the individual; G*Power Version 3.1.9.2, [47]). Although there is one study reporting an attrition rate of 52% after 6 months for participants of a weight loss intervention [48], many intervention trials targeting physical activity report attrition rates around 20% [50, 51] across 6 months to two years. Thus, we conservatively assume an attrition rate of 30%. This results in *N* = 116 (*n* = 29 per group) couples needed for appropriate powered analyses of the effect of the intervention across the three points of measurement.

For the analyses on the intraindividual level during the diary phase, it is not possible to run power analyses. This is due to the fact that these power calculations require detailed information on parameters from previous studies [49]. Because this study is the first to test effects of individual and dyadic action control on daily physical activity, this kind of power analysis cannot be reported. Therefore, this study will serve as a basis for future studies focusing on intraindividual associations of dyadic and individual action control interventions.

### Ethics

This study was approved by the institutional review board of the Faculty of Human Sciences of the University of Bern, 21 February 2012 (Reference number: 2011-12-36206).

## Discussion

Regular physical activity in accordance to the recommendations of the WHO can have important health benefits, especially in overweight and obese individuals. Changing one’s habitual physical inactivity, however, is a difficult endeavor. Common health behavior change theories focus almost exclusively on individual self-regulation. At the same time, there is research on social exchange processes (i.e., social support, social control) that demonstrate the importance of social network members with regard to health behavior change e.g., [32, 33]. One promising way to integrate social influence into the standard health behavior change approach is the dyadic conceptualization of individual regulation variables. For example, this has been done with implementation intentions e.g., [34, 35]. This study aims at targeting action control on a dyadic level by including couples and testing whether a partner-based dyadic action control intervention is more effective than an individual action control intervention compared with no-intervention control groups. The study uses an ecological momentary intervention [42] and combines macro- and micro-time assessments [38] in order to not only focus on longer-term effects of the intervention, but also to examine immediate intervention effects on a daily basis.

There were several issues that arose during the development of the study protocol and subsequently during the recruitment process and the implementation of the study. Regarding the study protocol and the planning of the intervention, one critical point is that the intervention comprises several components and that the two intervention groups are not merely different with regard to the ecological momentary intervention (i.e., the text message-based dyadic or individual action control intervention). Instead, the setting of the behavioral intentions also differs between the two groups in that target persons of the dyadic group form these intentions in collaboration with their partners and target persons of the individual group form these intentions alone. There were several reasons speaking in favor for this procedure. First, the dyadic action control intervention made it necessary to include partners into the goal setting process in order to have no logical problem when partners are sending text messages to the target persons regarding the intended increase in physical activity. Moreover, including the partners in the goal setting process might also increase the partners’ commitment to comply with the intervention protocol. Including the partners of the individual action control group also in the goal setting process, however, endangered introducing too much social influence into this group. Thus, the individual group participants completed the goal setting task on their own. Future studies might want to disentangle effects of joint goal setting and the text messages. For example, by including another experimental group that only engages in goal setting but does not receive text messages with action control content.

Another challenge was the decision as to whether providing smartphones to the participants or to let them use their own smartphones. The downside of the decision to provide smartphones to participating couples is that this might introduce quite an artificial component into the ecological momentary intervention design. For example, it is less natural to receive a text message from one’s partner on a smartphone provided by study personnel than on one’s own smartphone. However, there were several reasons why we decided in favor of providing smartphones. First, we did not want to exclude anyone who does not have an own smartphone. And second, with providing smartphones, we are able to control the intervention fidelity in terms of controlling if and when partners send the messages they were supposed to send to the target persons and if and when target persons received the text messages.

Finally, there is a trade-off between conducting a state-of-the-art ambulatory momentary assessment of physical activity and its antecedents by self-report and the danger of confounding effects of the ambulatory momentary assessment with the action control intervention. Instructing individuals to report all incidents of moderate-intensity physical activity over 10 minutes using the smartphones is likely to also trigger self-monitoring. Thus, the compromise we chose for this study was an objective ambulatory momentary monitoring by instructing participants to use accelerometers and assess the self-reported physical activity only once a day (in the hour before going to bed) in order to lower the impact of the diary method on our target variable action control. Moreover, in order to control for the effect of the diary on physical activity, we introduced control group II that has objective assessment of physical activity only, but no self-report in order to control for the potential effects of the self-reporting physical activity.

Another important point regarding the present study concerns the successful recruitment of couples meeting all the inclusion and none of the exclusion criteria and who are willing to partake in this intensive design. When it comes to couple studies, the first problem is that it always needs the consent of both partners to participate, which makes it much more difficult to find participants. When there are additionally relatively strict inclusion and exclusion criteria as is the case in the present study, the recruitment process can be very challenging. This needs to be kept in mind when evaluating the sample size of couple studies. In line with common standards we therefore recommend to abandon the strong focus on significance testing and to focus on effect sizes instead.

Despite these challenges outlined above in conducting studies with couples and applying ecological momentary interventions, we are convinced that this study will substantially further our knowledge with regard to social influences in health behavior change.

## Endnote

^a^One popular model of behavior change that explicitly integrates social support is the Transtheoretical Model of Behavior Change (TTM) [30]. In the TTM one of ten processes of change is “helping relationships” [30]. Helping relationships are defined as the support of others during behavioral change and is operationalized as a measure of *perceived* social support. Moreover, in the TPB [7] subjective norms are included as one predictor of intentions. Subjective norms, however, are defined as the subjective perception of expectations significant others have on the target person to change his/her behavior. Thus, social network partners are not explicitly integrated in the TPB.

## Electronic supplementary material

Additional file 1: Table S1: Full list of all text messages for all participants in original German wording and with English translation. (DOCX 15 KB)
