# Supplementary material for: A Dyadic Action Control Trial in Overweight and Obese Couples (DYACTIC)
Source: BMC Public Health. 2014 Dec 24;14:1321. doi: 10.1186/1471-2458-14-1321 (PMC4364646; doi:10.1186/1471-2458-14-1321)
Supplement: Supplementary file 1 — Additional file 1: Table S1: Full list of all text messages for all participants in original German wording and with English translation. (DOCX 15 KB) [file 12889_2014_7462_MOESM1_ESM.docx]

Additional file 1: Table S1. Full list of all text messages for all participants in original German wording and with English translation

| **no.** | **time** | **Experimental Group I & II (Target persons)*** | | **Experimental Group I (Partners)** | | **all others** |  |
| --- | --- | --- | --- | --- | --- | --- | --- |
|  |  | *Original* | *English translation* | *Original* | *English translation* | *Original* | *English translation* |
| 1 | 11:50 AM | Liebe/r xy, diese SMS soll eine kleine Erinnerung an deinen/Ihren Vorsatz sein, dich/sich täglich 30 Minuten zu bewegen. Herzliche Grüsse, [Partner/in] / die Studienleitung | Dear xy: This text message is a reminder on your intentions to be physically active for 30 minutes each day. Best wishes, [partner]/the study team | Liebe/r xy, bitte senden Sie Ihrem Partner/Ihrer Partnerin innerhalb der nächsten Stunde die **1. SMS** zu, und denken Sie heute Abend vor dem Zubettgehen an das Ausfüllen des Fragebogens. Herzlichen Dank, die Studienleitung | Dear xy, please send the **1st text message** within the next hour to your partner, and remember to fill in the diary on the smartphone tonight before going to bed. Many thanks, the study team | Liebe/r xy, denken Sie bitte heute Abend daran, den Fragebogen auf dem Smartphone auszufüllen, und zwar innerhalb der letzten Stunde bevor Sie zu Bett gehen. Herzlichen Dank, die Studienleitung | Dear xy, please remember to fill in the diary on the smartphone tonight, within one hour before going to bed. Many thanks, the study team |
| 2 | 2:15 PM | Liebe/r xy, welche der gefassten Vorsätze zur körperlichen Aktivität hast du/haben Sie heute schon ausgeführt? Herzliche Grüsse, [Partner/in] / die Studienleitung | Dear xy: Which of your intentions for your physical activity have you already carried out today? Best wishes, [partner]/the study team | Liebe/r xy, bitte senden Sie Ihrem Partner/Ihrer Partnerin innerhalb der nächsten Stunde die **2. SMS** zu, und denken Sie heute Abend vor dem Zubettgehen an das Ausfüllen des Fragebogens. Herzlichen Dank, die Studienleitung | Dear xy, please send the **2nd text message** within the next hour to your partner, and remember to fill in the diary before going to bed. Many thanks, the study team | Siehe 1. SMS | Same as 1. message |
| 3 | 10:55 AM | Liebe/r xy, falls du/Sie dein/Ihr Ziel von 30 Minuten Bewegung heute noch nicht erreicht hast/haben, lässt sich bestimmt noch eine gute Gelegenheit dazu finden! Herzliche Grüsse, [Partner/in] / die Studienleitung | Dear xy: If you haven‘t achieved your goal of 30 minutes physical activity today, there will certainly still be a good opportunity for it. Best wishes, [partner]/the study team | Liebe/r xy, bitte senden Sie Ihrem Partner/Ihrer Partnerin innerhalb der nächsten Stunde die **3. SM**S zu, und denken Sie heute Abend vor dem Zubettgehen an das Ausfüllen des Fragebogens. Herzlichen Dank, die Studienleitung | Dear xy, please send the **3rd text message** within the next hour to your partner, and remember to fill in the diary before going to bed. Many thanks, the study team | Siehe 1. SMS | Same as 1. message |
| 4 | 4:55 PM | Liebe/r xy, hast du deine/haben Sie Ihre Vorsätze zur täglichen Bewegung noch im Kopf? Herzliche Grüsse, [Partner/in] / die Studienleitung | Dear xy: Do you have your intentions for the daily activity still in mind? Best wishes, [partner]/the study team | Siehe oben mit Bezug auf **4.** SMS | Same as messages above refering to **4th** message | Siehe 1. SMS | Same as 1. message |
| 5 | 4:25 PM | Liebe/r xy, überlege dir/überlegen Sie sich kurz, wie oft du/Sie heute schon während mindestens 10 Minuten körperlich aktiv warst/waren. Wie viele Minuten fehlen noch? Herzliche Grüsse, [Partner/in] / die Studienleitung | Dear xy: Think about how often you have already engaged in at least 10 minutes of physical activity today. How many minutes are still missing? Best wishes, [partner]/the study team | Siehe oben mit Bezug auf **5.** SMS | Same as messages above refering to **5th** message | Siehe 1. SMS | Same as 1. message |
| 6 | 9:30 AM | Liebe/r xy, heute noch nicht so aktiv gewesen wie vorgenommen? Nutze/Nutzen Sie den Rest des Tages um dein/Ihr Ziel noch zu erreichen. Herzliche Grüsse, [Partner/in] / die Studienleitung | Dear xy: Haven't you been as active as intended today? Use the rest of the day to achieve your goal. Best wishes, [partner]/the study team | Siehe oben mit Bezug auf **6.** SMS | Same as messages above refering to **6th** message | Siehe 1. SMS | Same as 1. message |
| 7 | 11:10 AM | Liebe/r xy, Du hattest dir/Sie hatten sich vorgenommen, dich/sich jeden Tag körperlich zu betätigen, und zwar mindestens 3 x 10 Minuten am Stück. Herzliche Grüsse, [Partner/in] / die Studienleitung | Dear xy: You have intended to be physically active each day, namely at least 3x10 minutes at a stretch. Best wishes, [partner]/the study team | Siehe oben mit Bezug auf **7.** SMS | Same as messages above refering to **7th** message | Siehe 1. SMS | Same as 1. message |
| 8 | 11:55 AM | Liebe/r xy, warst du/waren Sie heute schon so aktiv, wie du dir/Sie sich das vorgenommen hast/haben? Herzliche Grüsse, [Partner/in] / die Studienleitung | Dear xy: Have you been as active today as you intended to be? Best wishes, [partner]/the study team | Siehe oben mit Bezug auf **8.** SMS | Same as messages above refering to **8th** message | Siehe 1. SMS | Same as 1. message |
| 9 | 3:05 PM | Liebe/r xy, versuch/versuchen Sie auch heute deinen/Ihren Vorsätzen für die tägliche Aktivität treu zu bleiben. Herzliche Grüsse, [Partner/in] / die Studienleitung | Dear xy: Try to stick with your intentions for your physical activity today. Best wishes, [partner]/the study team | Siehe oben mit Bezug auf **9**. SMS | Same as messages above refering to **9th** message | Siehe 1. SMS | Same as 1. message |
| 10 | 2:25 PM | Liebe/r xy, achte dich/achten Sie sich heute genau darauf, ob du/Sie lange und intensiv genug körperlich aktiv bist/sind. Herzliche Grüsse, [Partner/in] / die Studienleitung | Dear xy: Today, keep an eye closely on whether you are physically active long and intensive enough. Best wishes, [partner]/the study team | Siehe oben mit Bezug auf **10.** SMS | Same as messages above refering to **10th** message | Siehe 1. SMS | Same as 1. message |

Note: * In the experimental group I (dyadic action control group) the text messages are sent by the partners. The content is unchanged, but partners have the opportunity to adapt the message to their own Swiss-German dialect and personalize their greetings. In the experimental group II (individual action control group) the text messages are sent by the study personell.
